# APETALA2/ethylene responsive factor in fruit ripening: Roles, interactions and expression regulation

**DOI:** 10.3389/fpls.2022.979348

**Published:** 2022-08-18

**Authors:** Yanlei Zhai, Zhiyi Fan, Yuanyuan Cui, Xiaojiao Gu, Shangwu Chen, Huiqin Ma

**Affiliations:** ^1^College of Horticulture, China Agricultural University, Beijing, China; ^2^College of Food Science and Nutritional Engineering, China Agricultural University, Beijing, China

**Keywords:** AP2/ERF, fruit ripening, transcription regulation, protein interaction, phytohormone, epigenetic regulation

## Abstract

Insects and animals are attracted to, and feed on ripe fruit, thereby promoting seed dispersal. As a vital vitamin and nutrient source, fruit make up an indispensable and enjoyable component of the human diet. Fruit ripening involves a series of physiological and biochemical changes in, among others, pigmentation, chlorophyll (Chl) degradation, texture, sugar accumulation, and flavor. Growing evidence indicates that the coordinated and ordered trait changes during fruit ripening depend on a complex regulatory network consisting of transcription factors, co-regulators, hormonal signals, and epigenetic modifications. As one of the predominant transcription factor families in plants and a downstream component of ethylene signaling, more and more studies are showing that APETALA2/ethylene responsive factor (AP2/ERF) family transcription factors act as critical regulators in fruit ripening. In this review, we focus on the regulatory mechanisms of AP2/ERFs in fruit ripening, and in particular the recent results on their target genes and co-regulators. We summarize and discuss the role of AP2/ERFs in the formation of key fruit-ripening attributes, the enactment of their regulatory mechanisms by interaction with other proteins, their role in the orchestration of phytohormone-signaling networks, and the epigenetic modifications associated with their gene expression. Our aim is to provide a multidimensional perspective on the regulatory mechanisms of AP2/ERFs in fruit ripening, and a reference for understanding and furthering research on the roles of AP2/ERF in fruit ripening.

## Introduction

The fruit, which protects seeds and supports their development, begins ripening once the seeds mature. Ripe fruit encourages seed dispersal by attracting frugivorous animals, or through drying and dehiscence mechanisms (Forlani et al., 2019). As an essential source of sugars, vitamins, minerals, and antioxidants, fruit are desirable in the human diet. Fruit ripening is a complex process involving a series of physiological and biochemical changes in pigmentation, chlorophyll (Chl) degradation, texture, sugar accumulation, flavor formation, and so on, affecting fruit quality, postharvest life, and economic value (Chen et al., 2020).

Based on its ripening characteristics, fruit can be roughly categorized into climacteric and non-climacteric types. Climacteric fruit have concurrent peaks in respiration rate and ethylene production during ripening. Examples include apple, kiwi, tomato, banana, pear, peach, and mango. Non-climacteric fruit, such as strawberry, cherry, orange, and grape, do not display peaks in respiration rate or ethylene production during ripening (Gao et al., 2020). Although fruit development is co-regulated by a range of phytohormones, abscisic acid (ABA) and ethylene are generally considered to be the most critical ripening regulators. ABA can induce non-climacteric fruit ripening. Ethylene is essential for promoting climacteric fruit ripening (Chen et al., 2020).

Two ethylene-synthesis systems are found in fleshy fruit. System I contributes to the basal level of ethylene synthesis, and functions mainly during pre-ripening stages. System I is autoinhibitory, i.e., the perception of ethylene inhibits ethylene synthesis. In tomato (*Solanum lycopersicum* L.), *AMINOCYCLOPROPANE-1-CARBOXYLIC ACID SYNTHASE 1A* and *6* (*SlACS1A* and *SlACS6*) are involved in this process (Liu et al., 2015). System II is responsible for the burst of ethylene synthesis during climacteric fruit ripening and is autostimulated by ethylene signals; in tomato, this depends on *SlACS1A*, *SlACS2*, *SlACS4*, and *1-AMINOCYCLOPROPANE-1-CARBOXYLIC ACID OXIDASE 1* and *4* (*SlACO1* and *SlACO4*) (Forlani et al., 2019). The intensity of the ethylene signal in system II is closely related to the onset of ripening and the achievement of full ripening in tomato (Huang et al., 2022).

**FIGURE 1:**
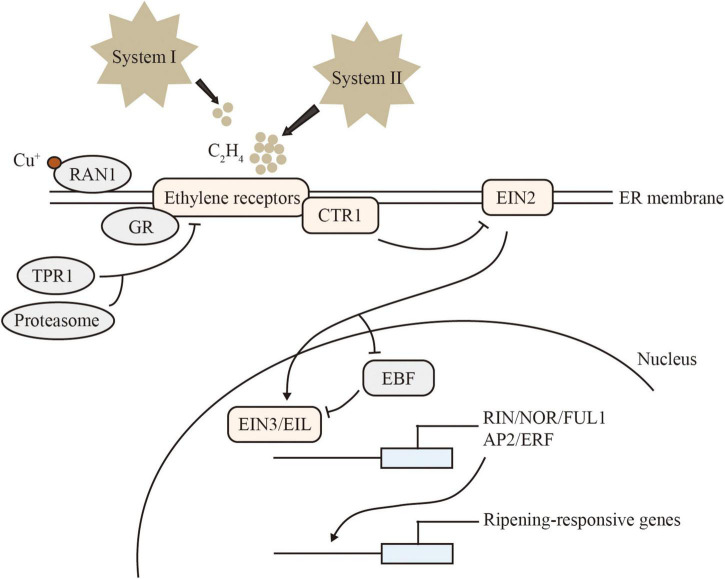
Model of ethylene signaling. CTR1 is a serine/threonine protein kinase that directly phosphorylates and inhibits EIN2 in the absence of ethylene. EIN2 contains multiple transmembrane domains at its N terminus and a cytoplasmic C terminus. In the presence of ethylene, the EIN2 C-terminal domain is cleaved and translocated into the nucleus. ETHYLENE INSENSITIVE3 BINDING F-BOX (EBF) proteins are responsible for targeting and degrading EIN3/EIL. EIN2 binds to and leads to the degradation of mRNAs encoding EBF1 and EBF2. In addition, EIN2 can regulate EIN3-dependent transcription. The accumulation and stabilization of EIN3/EIL lead to numerous transcriptional changes. Arrows represent activation, T-bars indicate repression.

Ethylene-signal transduction is conserved in climacteric and non-climacteric fruit (Figure 1). The receptors identified in *Arabidopsis*, including ETHYLENE RESPONSE 1/2 (ETR1/2), ETHYLENE RESPONSE SENSOR 1/2 (ERS1/2), and ETHYLENE INSENSITIVE 4 (EIN4), are located on the endoplasmic reticulum membrane. These receptors act as negative regulators of ethylene signaling. The tomato genome encodes more signal-transduction components than *Arabidopsis*, including 7 SlETRs, 4 SlCTRs (CONSTITUTIVE TRIPLE RESPONSE), 1 SlEIN2, 6 SlEILs (EIN3-Like), 4 SlEBFs (EIN3-BINDING F-BOX). *SlETR3*, *SlETR4*, and *SlETR7* are the main receptor genes expressed during tomato ripening (Liu et al., 2015). A single amino acid change in the N-terminal ethylene-binding pocket of SlETR3 results in impaired fruit ripening, known as the NEVER-RIPENING (NR) mutant (Wilkinson et al., 1995). The function of ETR1 receptor proteins has been reported to be regulated by GREEN-RIPE (GR), a homolog of the negative ethylene-response regulator REVERSION-TO-ETHYLENE SENSITIVITY1 (RTE1) of *Arabidopsis*, which can affect ethylene signaling in tomato (Barry and Giovannoni, 2006). RESPONSE TO ANTAGONIST 1 (RAN1) plays a vital role in the delivery of copper to the ethylene receptors, required for ethylene binding (Binder et al., 2010). The protein TETRATRICOPEPTIDE REPEAT (SlTPR1) interacts with the ethylene receptors NR and ETR1 to regulate ethylene and auxin responses (Lin et al., 2008).

Sensing ethylene inhibits receptor function, and relieves the inhibition of downstream pathways by the negative regulator CTR. Interestingly, a quantitative trait locus (QTL) containing a *CTR1-Like* gene and a putative DNA demethylase *REPRESSOR OF SILENCING 1* (*ROS1*) gene in melon triggers climacteric ripening on a non-climacteric background. CRISPR/Cas9 knockout mutants of *CTR1-Like* on the climacteric genetic background show significant advances in ethylene production and ripening initiation but without affecting other important traits, such as flesh firmness (Giordano et al., 2022). EIN2 is the central positive regulator of ethylene signaling. Loss-of-function mutants of SlEIN2 obtained by CRISPR/Cas9 gene editing exhibit fully impaired ethylene signaling. Cessation of ripening in *slein2* is partially rescued by *slebf1* (Huang et al., 2022). In the presence of ethylene, the EIN2 C-terminal domain is cleaved and translocated into the nucleus to activate the transcription factor EIN3 and its homolog EIL, which in turn induce the expression of downstream ethylene-responsive genes and transcription factors APETALA2/ethylene-responsive factor (AP2/ERF) (Ju and Chang, 2015).

The simplicity and conservation of ethylene signaling cannot explain the diversity and specificity of the resultant physiological responses. However, because AP2/ERFs act downstream of the ethylene-signaling pathway, the various ripening-related traits induced by ethylene can be explained by the diversity of these transcription factors (Liu et al., 2015, 2016).

## APETALA2/ethylene responsive factor family, characteristic domains and binding specificities

AP2/ERF is a large class of transcription factors that is found mainly in plants, characterized by an AP2/ERF domain involved in DNA binding. This domain is approximately 60–70 amino acids in length and consists of a three-stranded β-sheet and one α-helix almost parallel to the sheet; arginine and tryptophan are the pivotal residues in the β-sheet for DNA binding (Allen et al., 1998). The AP2 domain was first identified in the *Arabidopsis* AP2 protein, which functions in flower development (Jofuku et al., 1994). AP2/ERF is generally considered plant-specific. However, homologs of the AP2 domain have been identified in the cyanobacterium *Trichodesmium erythraeum*, the ciliate *Tetrahymena thermophila*, and the viruses *Enterobacteria phage Rb49* and *Bacteriophage Felix 01*, suggesting that plant AP2/ERFs may originate from horizontal transfer of HNH-AP2 endonuclease from bacteria or viruses via transposition and homing processes (Magnani et al., 2004).

Thanks to increasingly available genome data, hundreds of AP2/ERF genes have been identified from different plants, such as *Arabidopsis thaliana* (147, Nakano et al., 2006), tomato (*Solanum lycopersicum*; 146, Pirrello et al., 2012), apple (*Malus* × *domestica*; 259, Girardi et al., 2013), grapevine (*Vitis vinifera*; 132, Zhuang et al., 2009), pineapple (*Ananas comosus* L. Merr; 97, Zhang et al., 2021), longan (*Dimocarpus longan* Lour.; 125, Zhang S. et al., 2020), peach (*Prunus persica*; 131, Zhang et al., 2012), Chinese jujube (*Ziziphus jujuba* Mill.; 119, Zhang and Li, 2018), sweet orange (*Citrus sinensis*; 108, Ito et al., 2014), and others. Systematic analyses indicate that whole-genome duplication events, segmental duplication, and tandem duplication have contributed to the expansion of the AP2/ERF family in plants (Zhuang et al., 2009; Zhang et al., 2021). Duplicated genes evolve overlapping or distinct regulatory functions (Shoji and Yuan, 2021; Zhai et al., 2021), and expansion of the AP2/ERF family has brought about functional differentiation, leading to crucial roles for AP2/ERFs in a wide range of biological and physiological processes such as morphogenesis, defense responses, signal transduction, organ senescence, and metabolite regulation (Feng et al., 2020; Gao et al., 2020).

The *Arabidopsis* AP2/ERF superfamily is divided into four main categories: ERF (first discovered in Ethylene-Responsive Element-Binding proteins (EREBPs), AP2, Related to Abscisic Acid Insensitive 3/Viviparous 1 (RAV), and soloists (a few unclassified factors) (Nakano et al., 2006; Feng et al., 2020). The AP2 family usually contains two repeated AP2/ERF domains. In addition to one AP2/ERF domain, the RAV family also has one B3 domain, a DNA-binding domain that is conserved in other transcription factors, such as VP1/ABI3. The ERF family contains a single AP2/ERF domain. According to the conserved amino acid residues at positions 14 and 19 of this domain, the ERF family can be further divided into the ERF subfamily and the C-Repeat/Dehydration-Responsive Element Binding factors (CBF/DREB) subfamily (Sakuma et al., 2002). Based on the phylogenetic relationship and conserved motif characteristics, the ERF subfamily can be further divided into 12 phylogenetic groups, namely I to X, VI-L, and Xb-L (Nakano et al., 2006). In tomato, the ERF subfamily members are distributed into A–J groups (Liu et al., 2016).

AP2/ERF proteins bind directly to *cis*-acting elements on the target gene’s promoter, such as dehydration-responsive element/C-repeat element (DRE/CRT) with core sequence CCGAC, GCC box with core sequence AGCGCCC, and some other GC-rich motifs. The divergent DNA-binding specificities are associated with the amino acid residues of the AP2 domain, which affect the interaction of AP2/ERF proteins with DNA bases and phosphate backbones (Shoji et al., 2013). Furthermore, the AP2 and B3 domains of the RAV subfamily specifically recognize CAACA and CACCTG motifs, respectively (Kagaya et al., 1999). It has been reported that some ERFs can bind the ATCTA motif (Welsch et al., 2007) and vascular system-specific and wound-responsive *cis*-element (VWRE) (Sasaki et al., 2007). Nucleotides flanking the *cis*-element also enhance or weaken the binding affinity of AP2/ERF, thereby conferring different AP2/ERF binding specificities to target genes (Pirrello et al., 2012).

AP2/ERFs perform transcriptional activation or repression functions, mainly depending on the functional domain other than the DNA-binding domain. The hydrophobic amino acid-containing ERF-Associated Amphiphilic Repression (EAR) motif is the most dominant active repression domain identified in plants. It is widely found in AP2/ERFs with transcriptional repression function, and is mainly located in the C-terminal region of the protein, with conserved sequences LxLxL or DLNxxP (Ohta et al., 2001; Kagale et al., 2010). Moreover, a B3 Repression Domain (BRD) was identified in the RAV subfamily, containing the R/KLFGV conserved motif (Ikeda and Ohme-Takagi, 2009). The activation domains usually exhibit sequence divergence. The acidic amino acids are thought to be involved in transcriptional activation (Liu et al., 1999). An EDLL motif has been identified as a strong activation domain in group IX of the ERF family, containing several acidic amino acids spaced around hydrophobic leucines (Tiwari et al., 2012).

An N-terminal conserved MCGGAII/L domain has been identified in group VII of the ERF subfamily, involved in oxygen-sensing and N-end rule pathway-dependent protein degradation. Post-translational modifications of this domain can affect the activity of the transcription factors, thereby regulating the expression of core hypoxia responsive genes (Gibbs et al., 2011; Licausi et al., 2011). Group E members of ERF in tomato (corresponding to group VII in *Arabidopsis*) exhibit tight ripening-related expression and are thought to play a prominent role in ethylene- and RIN (RIPENING-INHIBITOR)/NOR (NON-RIPENING)-dependent ripening (Liu et al., 2016).

## Role in fruit ripening

AP2/ERFs play critical roles in fruit ripening (Table 1). In tomato, 55 ERF subfamily members showed ripening-related expression patterns, 27 upregulated and 28 downregulated (Liu et al., 2016). AP2/ERFs are involved in textural changes, pigmentation, Chl degradation, and flavor formation via their regulation of downstream ripening-related genes.

**TABLE 1:** AP2/ERF regulation of different ripening-related traits.

Regulated traits	Species	Gene name	Target genes	Interacting *cis*-element	Function	References
Softening	Banana (*Musa acuminata*)	*MaDEAR1*	*MaEXP1/3, MaPG1, MaXTH10, MaPL3, MaPME3*	DRE/CRT motif	Repressor	Fan et al., 2016
	Papaya (*Carica papaya* L.)	*CpERF9*	*CpPME1/2, CpPG5*	GCC box	Repressor	Fu et al., 2016
	Apple (*Malus* × *domestica*)	*MdERF4*	*MdERF3*	DRE/CRT motif	Repressor	Hu et al., 2020, 2022a
	Persimmon (*Diospyros kaki*)	*DkERF8/16/19*	*DkXTH11, DkEXP4, DkXTH9*	GCC box, DRE/CRT motif	Activator	Wang et al., 2017; He et al., 2020
	Peach (*Prunus persica*)	*PpeERF2*	*PpePG1*	DRE/CRT motif	Repressor	Wang et al., 2019a
	Banana (*Musa acuminata*)	*MaERF11*	*MaEXP2, MaEXP7, MaEXP8*	GCC box	Repressor	Han et al., 2016
	Tomato (*Solanum lycopersicum*)	*SlERF.F12*	*SlPG2a, SlPL*	GCC box, DRE/CRT motif	Repressor	Deng et al., 2022
	Banana (*Musa acuminata*)	*MaERF9*	*MaEXP1/2/3/5, MaXET7, MaPG1, MaPME3, MaPL2*	GCC box	Activator	Feng et al., 2016
	Peach (*Prunus persica*)	*PpERF4*	*PpPG1*	DRE/CRT motif	Activator	Wang et al., 2021
	Banana (*Musa acuminata*)	*MaDREB2*	*MaEXP1, MaEXP3, MaEXP5, MaXET3, MaXET7*	(A/G)CC(G/C)AC	Repressor	Kuang et al., 2017
Chlorophyll degradation	Apple (*Malus* × *domestica*)	*MdERF17*	*MdPPH, MdNYC*	CACGGT, CACGTG	Activator	Han et al., 2018; Wang et al., 2022
	Citrus fruit (*Citrus reticulata*)	*CitERF6, CitERF13*	*CitNYC, CitPPH*	DRE/CRT motif	Activator	Li et al., 2019
	Grape (*Vitis vinifera*)	*VvERF17*	*VvNOL, VvPPH, VvPAO, VvRCCR*	CAACA, CACGTG	Activator	Lu et al., 2022
Anthocyanin and flavonoid accumulation	Apple (*Malus* × *domestica*)	*MdAP2_1a*	*MdMYB10*	Not mentioned	Activator	Ding et al., 2022
	Apple (*Malus* × *domestica*)	*MdERF109*	*MdCHS, MdUFGT, MdbHLH3*	GCC box	Activator	Ma et al., 2021
	Apple (*Malus* × *domestica*)	*MdERF1B*	*MdMYB9, MdMYB11*	RAA (CAACA) motif	Activator	Zhang J. et al., 2018
	Pear (*Pyrus* spp.)	*PpERF105*	*PpMYB140*	Not mentioned	Activator	Ni et al., 2021
	Strawberry (*Fragaria* × *ananassa*)	*FaRAV1*	*FaMYB10, FaCHS, FaF3H, FaDFR, FaGT1*	RAA (CAACA) motif	Activator	Zhang Z. et al., 2020
	Citrus fruit (*Citrus reticulata*)	*CitERF32, CitERF33, CitRAV1*	*CitCHIL1*	CGCCGC	Activator	Zhao et al., 2021
Carotenoid accumulation	Apple (*Malus* × *domestica*)	*MdAP2-34*	*MdPSY2-1*	DRE/CRT motif	Activator	Dang et al., 2021
	Citrus fruit (*Citrus sinensis*)	*CsERF061*	*LCYb2, PSY1, PDS, CRTISO, LCYb1, BCH, ZEP, NCED3, CCD1, CCD4*	ERE motif (AATTCAAA), DRE/CRT motif, GCC box	Activator	Zhu et al., 2021
Aroma accumulation	Peach (*Prunus persica*)	*PpERF61*	*PpTPS1/3, PpbHLH1*	DRE/CRT motif	Activator	Wei et al., 2022
	Sweet orange (*Citrus sinensis* Osbeck)	*CitERF71*	*CitTPS16*	ACCCGCC and GGCGGG motifs	Activator	Li X. et al., 2017
	Sweet orange (*Citrus sinensis*)	*CitAP2.10*	*CsTPS1*	Not mentioned	Activator	Shen et al., 2016
	Banana (*Musa acuminata*)	*MaDREB2*	*MaADH1, MaPDC*	(A/G)CC(G/C)AC	Repressor	Kuang et al., 2017
	Banana (*Musa acuminata*)	*MaERF9*	*MaCAT, MaPDC*	GCC box	Activator	Feng et al., 2016

### Cell wall modification

The remarkable change in fruit texture at ripening is characterized by a process of remodeling cell wall structure and composition, coordinated by a series of cell wall-modifying enzymes. Among them, POLYGALACTURONASE (PG), PECTIN METHYLESTERASE (PME) and PECTATE LYASE (PL) are related to the metabolism of pectin, and XYLOGLUCAN ENDOTRANSGLUCOSYLASE/HYDROLASE (XTH) is related to xyloglucan and hemicellulose metabolism (Tucker et al., 2017). Recent studies have found that AP2/ERFs regulate the transcription of several cell wall-modifying genes. In persimmon (*Diospyros kaki*), DkERF8/16/19 bind to the DRE/CRT element of *DkXTH9*’s promoter and activate its transcription (Wang et al., 2017). DkERF8 and DkERF16 also activate *DkXTH11* and *EXPANSIN 4* (*DkEXP4*), respectively, *via* binding to their promoters (He et al., 2020).

In peach, PpeERF2 was found to bind directly to the *PpePG1* promoter by yeast one-hybrid (Y1H) and electrophoretic mobility shift assay (EMSA), and further found to repress its expression by *Agrobacterium* infiltration (Wang et al., 2019a). During banana (*Musa acuminata*) ripening, MaDEAR1 represses the expression of *MaEXP1/3*, *MaPG1*, *MaXTH10*, *MaPL3*, and *MaPME3* (Fan et al., 2016). In papaya (*Carica papaya* L.), CpERF9 binds to the GCC box of the *CpPME1/2* and *CpPG5* promoters and represses their expression (Fu et al., 2016, 2021). Notably, members of the F group characterized by the EAR motif have been widely found to inhibit cell wall-modifying gene expression. A recent study hypothesized that the rate of fruit softening depends on the balance between the ERF.F repressors and other activators. Another possibility is that ERF.F is inhibited after ripening begins, subsequently releasing the inhibition of cell wall-modification genes (Shi et al., 2022).

The G–C mutation in the EAR motif of apple *MdERF4* impairs its transcriptional repression of *MdERF3*, thereby promoting ethylene production and loss of fruit firmness (Hu et al., 2020). Watermelon (*Citrullus lanatus*) ClERF4, associated with variations in fruit peel firmness, was identified via a combinatorial genetic map and bulk segregant analysis. An 11-bp indel and the neighboring single-nucleotide polymorphism in *ClERF4* contributed to differences in rind hardness and cracking resistance (Liao et al., 2020). These findings suggest that ERF allelic mutations play an important role in textural fruit traits.

Cell wall modification contributes not only to softening but also to tissue expansion and growth. XTH is thought to be involved in maintaining the structural integrity of the cell wall during fruit development, while contributing to softening at the onset of ripening (Miedes and Lorences, 2009). This dual role is related to the dual biochemical function of XTH, with different members acting as xyloglucan endotransglucosylase (XET) or xyloglucan endohydrolase (XEH) (Rose et al., 2002). The expression and activity of several XTHs differ at different developmental stages of the fruit and are regulated by ethylene (Goulao et al., 2007; Tucker et al., 2017). It is assumed that AP2/ERFs participate in the change in cell wall state from fruit development through the onset of ripening to full ripeness by differentially regulating cell wall-modifying enzymes.

### Color

Fruit color development at ripening depends mainly on the contents of flavonoids, carotenoids, and Chl (Wang et al., 2022). AP2/ERFs participate in fruit degreening by regulating Chl degradation. In citrus (*Citrus reticulata*), ethylene-induced CitERF6 and CitERF13 trigger Chl degradation by directly activating the expression of *NON-YELLOW COLORING* (*CitNYC*) and *PHEOPHYTINASE* (*CitPPH*), as identified by dual-luciferase and Y1H assay (Li et al., 2019). In apple, mutations in the coding region of *MdERF17* affect peel degreening. Different numbers of serine repeats affect the transcription-regulatory activity of MdERF17 mutant alleles on Chl degradation-related genes, including *MdPPH* and *MdNYC*, as observed in a dual-luciferase reporter assay (Han et al., 2018). VvERF17 promotes Chl degradation in grape berry by activating several Chl catabolic genes, including *CHLOROPHYLL-B-REDUCTASE* (*VvNOL*), *VvPPH*, *PHEOPHORBIDE*α *OXYGENASE* (*VvPAO*), and *RED CHLOROPHYLL CATABOLITE REDUCTASE* (*VvRCCR*) (Lu et al., 2022).

Ethylene has been reported to regulate anthocyanin biosynthesis in many fruit (Ni et al., 2021). Ethylene treatment accelerates apple peel coloration, during which MdEIL1 activates *MdMYB1* transcription. MdMYB1 promotes anthocyanin biosynthesis and activates the expression of *MdERF3* (a key regulator of ethylene synthesis), providing positive feedback for ethylene signaling and fruit coloration (An et al., 2018). MdERF1B contributes to apple coloration by activating the transcription of *MdMYB9* and *MdMYB11* (Zhang J. et al., 2018). In strawberry (*Fragaria* × *ananassa*), FaRAV1 activates the expression of *FaMYB10*, an essential gene for anthocyanin biosynthesis, by binding to its promoter. FaRAV1 can also directly promote the transcription of *CHALCONE SYNTHASE* (*FaCHS*), *FLAVANONE 3-HYDROXYLASE* (*FaF3H*), *DIHYDROFLAVONOL-4-REDUCTASE* (*FaDFR*) and *3-GLYCOSYLTRANSFERASE* (*FaGT1*), and enhance anthocyanin accumulation (Zhang Z. et al., 2020). Ethylene-induced PpERF105 activates the transcription of *PpMYB140*, which is a repressor of anthocyanin biosynthesis in pear (*Pyrus* spp.) (Ni et al., 2021).

Carotenoids are another major pigment component. The expression of *MdAP2-34* promotes carotenoid accumulation in apple callus and fruit. MdAP2-34 can directly activate the expression of *PHYTOENE SYNTHASE 2* (*MdPSY2-1*), which is a key gene in carotenoid biosynthesis (Dang et al., 2021). In citrus (*C. sinensis*) fruit, ethylene-induced CsERF061 activates the expression of 10 carotenoid-related genes—*LYCOPENE*β*- CYCLASE* (*LCYb2*), *PSY1*, *PHYTOENE DESATURASE* (*PDS*), *CAROTENOID ISOMERASE* (*CRTISO*), *LCYb1*, β*-CAROTENE HYDROXYLASE (BCH)*, *ZEAXANTHIN EPOXIDASE* (*ZEP*), *9-CIS-EPOXYCAROTENOID DIOXYGENASE* (*NCED3*), *CAROTENOID CLEAVAGE DIOXYGENASE* (*CCD1*), and *CCD4*, thus enhancing carotenoid synthesis through multitarget regulation (Zhu et al., 2021).

### Aroma

Volatile esters are the main components of the aroma of strawberry, apple, banana, and other fruit. Alcohol acyltransferase (*AAT*) is considered the key gene in ester biosynthesis and has a significant effect on aroma formation. Ethylene is thought to affect ester biosynthesis in apple by regulating *AAT* (Defilippi et al., 2005). Several ERF genes have been shown to be associated with the expression of *AAT* in strawberry via transcriptome analysis and weight co-expression network analysis (WGCNA). Overexpression of FveERF indeed activates *AAT* gene expression and ester accumulation in strawberry (*Fragaria vesca*) fruit (Li et al., 2020). Ethylene treatment increased volatile production in banana fruit, consistent with upregulation of volatile biosynthetic genes at the transcriptional level, including *PYRUVATE DECARBOXYLASE* (*PDC*) and *ALCOHOL DEHYDROGENASE* (*ADH*) (Yang et al., 2011). Chromatin immunoprecipitation (ChIP)-qPCR and EMSA revealed that banana MaDREB2 binds directly to the promoters of *MaADH1* and *MaPDC* (Kuang et al., 2017).

The monoterpene E-geraniol and sesquiterpene (+)-valencene are important volatile compounds for flavor formation in sweet orange. CitERF71 binds directly to the promoter of *TERPENE SYNTHASE 16* (*CitTPS16*) and activates its expression to regulate E-geraniol biosynthesis in citrus fruit (Li X. et al., 2017). Dual-luciferase assays indicated that ethylene-induced CitAP2.10 transactivates *CsTPS1*, regulating (+)-valencene biosynthesis (Shen et al., 2016). Transcriptome and biochemical analyses of AP2/ERF PaWRI1-2 revealed functions associated with fatty acid accumulation in avocado fruit (*Persea americana* Mill.), affecting fruit quality and nutritional value (Ge et al., 2021). In apple pericarp, MdERF3 binds to the DRE motif of α-FARNESENE SYNTHASE (*MdAFS*) and activates its expression to promote biosynthesis of α-farnesene, which is related to insect attraction and plant defense (Wang et al., 2020). In banana, the transcriptional activator MaERF9 interacts physically with the transcriptional repressor DNA BINDING WITH ONE FINGER 23 (MaDOF23) to antagonistically regulate the expression of two aroma-related genes, *BRANCHED-CHAIN AMINO ACID TRANSAMINASE* (*MaCAT*) and *PYRUVATE DECARBOXYLASE* (*MaPDC*) (Feng et al., 2016). These findings suggest that AP2/ERFs play a vital role in regulating fruit ripening-related aroma development.

## APETALA2/ethylene responsive factor interacts with other proteins in regulating fruit ripening

Transcription factors can interact with many different proteins, including other transcription factors, co-regulators, and components of basal transcription complexes, resulting in effects on transcription factor cell localization, protein stability, protein–protein interactions, and DNA binding (Whitmarsh and Davis, 2000). Physical interactions between AP2/ERFs and other proteins have been widely reported, involving processes such as cell wall modification, anthocyanin synthesis, and flavor accumulation. In persimmon, DkERF8 and DkERF16, previously mentioned as direct regulators of cell wall modifications, interact with the DkNAC9 protein. Moreover, DkNAC9 binds directly to the promoter of *ENDO-1,4-*β*-D-GLUCANASE* (*DkEGase1*) and activates its expression, revealing a synergistic role for ERF and NAC in cell wall remodeling (Wu et al., 2020).

In apple, MdERF1B interacts with MdMYB9, MdMYB1, and MdMYB11 (all related to apple anthocyanin biosynthesis) proteins in yeast two-hybrid (Y2H), pull-down, and bimolecular fluorescence complementation (BiFC) assays, and binds to their promoters (Zhang J. et al., 2018). MdERF38 interacts with MdMYB1, which enhances the binding of MdMYB1 to the promoters of anthocyanin-biosynthesis genes (An et al., 2020). PyERF3 affects anthocyanin accumulation in pear by interacting with PyMYB114 (Yao et al., 2017). Pp4ERF24 and Pp12ERF96 interact with PpMYB114 and enhance PpMYB114-mediated *UDP GLUCOSE:FLAVONOID 3-O-GLUCOSYLTRANSFERASE* (*PpUFGT*) expression (Ni et al., 2019). CitRAV1 interacts with CitERF33 in citrus, enhancing the transcriptional activation of *CHALCONE ISOMERASE* (*CitCHIL1*) by CitERF33 and promoting flavonoid accumulation (Zhao et al., 2021). These findings provide insight into how ethylene regulates anthocyanin synthesis. In strawberry, FaERF#9 indirectly activates the expression of *QUINONE OXIDOREDUCTASE* (*FaQR*) by forming an ERF–MYB complex with FaMYB98, thus promoting the biosynthesis of 4-hydroxy-2,5-dimethyl-3(2H)-furanone and flavor accumulation (Zhang Y. et al., 2018).

AP2/ERFs with the EAR motif respond to various biotic and abiotic stresses in plants, including salinity, wounding, low temperature, drought and pathogens (Dong and Liu, 2010; Lu et al., 2011; Dong et al., 2012, 2015). EAR is the most common transcriptional-repression motif identified in plants. A total of 219 candidate transcriptional regulators with EAR motifs were identified in *Arabidopsis*, belonging to 21 different transcription-regulator families. The LxLxL type accounted for most of them (72%) (Kagale et al., 2010). EAR motif-mediated transcriptional repression is one of the principal mechanisms of gene regulation in plants, mainly through physical interactions with other co-repressors, including SWITCH INDEPENDENT 3 (SIN3, Song et al., 2005), SIN3 ASSOCIATED POLYPEPTIDE 18 (SAP18, Hill et al., 2008), TOPLESS/TOPLESS-RELATED (TPL/TPR), and histone deacetylases (Deng et al., 2022).

In apple, MdTPL4 and HISTONE DEACETYLASE 19 (MdHDA19) are recruited by the EAR motif-containing MdERF4, and the protein complex inhibits *MdACSa* expression, thereby affecting ethylene synthesis and fruit ripening (Hu et al., 2022a). In tomato, SlERF.F12 (with both types of EAR motifs) represses the expression of multiple ethylene-synthesis and cell wall-degradation genes by recruiting TPL2 and HDA1/HDA3 (Deng et al., 2022). In banana, MaERF11, with the EAR motif, recruits MaHDA1 and represses the expression of a range of ripening-related genes through histone deacetylation (Han et al., 2016). The EAR motif works in conjunction with both TPL and histone deacetylase, supporting a model in which it mediates transcriptional repression by recruiting chromatin remodelers. Moreover, conserved residues adjacent and integral to the EAR motif are involved in the post-translational regulation (Kagale et al., 2010). For example, serine and threonine residues within and around the EAR motifs are regulated by phosphorylation (Kagale and Rozwadowski, 2010).

Post-translational modifications are essential regulatory mechanisms in plants that respond to extracellular signaling molecules and environmental changes, resulting in rapid changes in protein status and transcriptional activity (Whitmarsh and Davis, 2000). In apple, MdERF17 interacts with and is phosphorylated by MAP KINASE4 (MdMPK4-14G). MdERF17 mutants with different numbers of serine repeats display diverse phosphorylation profiles. Phosphorylation of MdERF17 by MdMPK4-14G is necessary to regulate peel Chl degradation (Wang et al., 2022). Moreover, BTB AND TAZ DOMAIN PROTEIN MdBT2, with ubiquitinase activity, accelerates MdERF38 protein degradation and reduces MdERF38-promoted anthocyanin biosynthesis in apple coloration (An et al., 2020).

## APETALA2/ethylene responsive factor in plant hormone signaling

A complex network of hormonal crosstalk coordinates fruit ripening. Early research on ripening-related hormones focused on ethylene, especially in climacteric fruits. Ethylene treatment promotes a climacteric rise in banana, and upregulation of MaERF9 and downregulation of MaERF11 are thought to contribute to fruit-ripening regulation (Xiao et al., 2013). In fig (*Ficus carica L.*), exogenous ethephon treatment results in accelerated fruit ripening; downregulation of the high expression of AP2/ERFs was found in ethephon-treated fruit 2 and 4 days after the treatment except for two genes, and the ethephon-induced AP2/ERF repression generally ended 6 days after the application (Cui et al., 2021).

AP2/ERFs are not only regulated by ethylene; they also regulate ethylene synthesis (Table 2). In pear, ERF24 binds directly to the promoter of *ACO54* and activates its expression, while overexpression of *ACO54* also increases the expression of *ERF24* (Hao et al., 2018). In apple, MdERF2 binds to the promoter of *MdERF3* and represses its expression, thereby reducing MdERF3-promoted transcription of *MdACS1*. Moreover, MdERF2 binds directly to the promoter of *MdACS1* and represses its transcription, resulting in inhibition of ethylene biosynthesis (Li et al., 2016). MdMYB1 can promote the expression of *MdERF3*, revealing a synergistic regulation mechanism (An et al., 2018). In banana, MaERF11 binds to the promoters of *MaACS1* and *MaACO1* and represses their expression, whereas MaERF9 activates *MaACO1* transcription (Xiao et al., 2013). In peach, ethylene-induced PpERF.A16 enhances ethylene biosynthesis by directly activating the expression of *PpACS1* and *PpACO1*. At the same time, *PpERF.A16* is transcriptionally regulated by PpNAC.A59 (Guo et al., 2021).

**TABLE 2:** AP2/ERF regulation of hormone signaling.

Species	Gene name	Target genes	Interacting *cis*-element	Function	References
Apple (*Malus* × *domestica*)	*MdERF2, MdERF3*	*MdERF3, MdACS1, MdACS3a*	DRE/CRT motif	Activator and repressor	Li et al., 2016; Yue et al., 2020
Banana (*Musa acuminata*)	*MaERF9, MaERF11*	*MaACS1, MaACO1*	Not mentioned	Activator and repressor	Xiao et al., 2013
Peach (*Prunus persica*)	*PpERF.A16*	*PpACS1, PpACO1*	GCCGCC, GGCGTC	Activator	Guo et al., 2021
Pear (*Pyrus* spp.)	*ERF24*	*ACO54*	Not mentioned	Activator	Hao et al., 2018
Persimmon (*Diospyros kaki*)	*DkERF18*	*DkACS2*	DRE/CRT motif	Activator	He et al., 2020
Banana (*Musa acuminata*)	*MaERF11*	*MaACO1*	GCC box	Repressor	Han et al., 2016
Tomato (*Solanum lycopersicum*)	*SlERF.F12*	*SlACS2, SlACS4*	GCC box, DRE/CRT motif	Repressor	Deng et al., 2022
Peach (*Prunus persica*)	*PpERF3*	*PpNCED2/3*	DRE/CRT motif	Activator	Wang et al., 2019b
Peach (*Prunus persica*)	*PpeERF2*	*PpeNCED2, PpeNCED3*	DRE/CRT motif	Repressor	Wang et al., 2019a
Peach (*Prunus persica*)	*PpERF4*	*PpIAA1, PpACO1, PpNCED2, PpNCED3*	GCC box, DRE/CRT motif	Activator	Wang et al., 2021
Pear (*Pyrus ussuriensis*)	*PuERF2*	*PuGH3.1*	DRE/CRT motif	Activator	Yue et al., 2019
Tomato (*Solanum lycopersicum*)	*SlERF.B3*	*SlIAA27, SlETR6, SlERF.C3, SlERF.D2, SlERF.F5, SlERF.F4*	GCC box, DRE/CRT motif	Activator	Liu et al., 2013, 2018

Several ERFs regulate ethylene synthesis and fruit ripening in tomato. SlERF6 silencing by RNAi increases ethylene levels and carotenoid content during fruit ripening (Lee et al., 2012). APETALA2a (AP2a) negatively regulates fruit ripening. RNAi repression of *SlAP2a* results in overproduction of ethylene and altered carotenoid accumulation (Chung et al., 2010). Overexpression of *SlERF.B3-SRDX* (a chimeric dominant repressor version) shows contrasting effects on fruit ripening, resulting in delayed ripening onset, enhanced ethylene production and fruit softening, and reduced carotenoid biosynthesis. Consistent with the phenotypes, the expression of ripening-related genes is highly induced, such as ethylene-synthesis genes *ACS2*, *ACS4* and *ACO1*, softening gene *PG2A*, developmental regulators *RIN*, *NOR* and *CNR*, and a set of *ERF* genes (Liu et al., 2013, 2014).

The regulatory role of ethylene in fruit ripening is affected by the signaling network formed by interactions with other hormones and transcription factors (Kou et al., 2021), the latter serving as correlated connectors for the crosstalk (Li et al., 2022). In tomato, the ethylene-responsive factor SlPti4 regulates fruit ripening by affecting ABA metabolism and signaling, and silencing *SlPti4* results in increased ABA accumulation and decreased ethylene release (Sun et al., 2018). In peach, PpeERF2 binds to and represses the transcription of two ABA-biosynthesis genes, *PpeNCED2/3* (Wang et al., 2019a). In contrast, PpERF3 transactivates *PpNCED2/3*, thereby increasing ABA biosynthesis (Wang et al., 2019b).

Crosstalk between auxin and ethylene is necessary for fruit development and ripening. Downregulation of *AUXIN-RESPONSIVE FACTOR 2* (*ARF2*) in tomato, a downstream factor in auxin signaling, results in ripening defects, reduced climacteric ethylene synthesis and delayed ripening. SlARF2-RNAi lines showed significant downregulation of ethylene receptor genes *ETR3* and *ETR4*, and 12 and 5 AP2/ERFs were found downregulated and upregulated, respectively, in the tomato fruit (Hao et al., 2015). In apple, 4 mM auxin naphthaleneacetic acid treatment before the commercial harvest stage induced ethylene synthesis and fruit ripening; MdARF5 activated the transcription of *MdERF2*, two *MdACS*s and *MdACO1* by directly binding to their promoters (Yue et al., 2020). Ethephon treatment reduced free indole acetic acid (IAA) content in pear; during this process, PuERF2 activated the transcription of *GRETCHEN HAGEN 3* (*PuGH3.1*), and PuGH3.1 conjugated free IAA to inactive IAA-amide (Yue et al., 2019). In tomato, SlERF.B3 integrates ethylene and auxin signaling by directly binding to the promoter of *SlIAA27*; ectopic expression of *SlERF.B3* results in phenotypes similar to those of *SlIAA27*-downregulated lines, such as elongated primary root and remarkably increased lateral root formation (Liu et al., 2018). A positive feedback loop of ripening regulation was revealed in peach: PpIAA1, which can be upregulated by both ethylene and auxin, promoted the expression of *PpACS1* and *PpNCED2/3*. PpERF4 activated the transcription of *PpIAA1* and physically interacted with PpIAA1 protein, thereby enhancing the latter’s transcription-activation ability (Wang et al., 2021).

Although most studies on jasmonate (JA) have focused on responses to biotic and abiotic stresses, recent studies have revealed its involvement in fruit-ripening regulation. In apple, the transcription factor MYELOCYTOMATOSIS-RELATED PROTEINS 2 (MdMYC2), involved in JA signaling, binds directly to the promoters of *MdERF3*, *MdACS1*, and *MdACO1* and activates their expression. MdMYC2 also physically interacts with MdERF2, thereby reducing the latter’s transcriptional repression of *MdERF3* and *MdACS1*, and increasing ethylene production (Li T. et al., 2017). MdERF4 physically interacts with JASMONATE ZIM-DOMAIN (JAZ) to form a repressor complex that acts as a molecular link between ethylene and JA signaling (Hu et al., 2022b).

## Regulation of APETALA2/ethylene responsive factor expression by epigenetic mechanisms

Epigenetic modifications affect mainly DNA methylation, as well as the methylation, acetylation, phosphorylation, and ubiquitination status of histones (Giovannoni et al., 2017). Examination of the role of DNA methylation during ripening in several fruit crops has revealed the occurrence of global epigenome reprogramming. For example, 5-azacytidine, a general inhibitor of DNA (cytosine-5) methyltransferase, promotes early ripening of immature tomatoes. Using whole-genome bisulfite sequencing, 52,095 differentially methylated regions (DMRs) were identified, representing 1% of the genome. During tomato fruit ripening, DNA methylation levels at the 5′ end of genes generally declined across the genome. The demethylation events promoted binding of a master ripening-related regulator, RIN, to promoters of a series of ripening genes, including several AP2/ERFs (Zhong et al., 2013).

Overall loss of DNA methylation was also found during strawberry ripening. A total of 2766 DMRs were identified, of which 466 were hypermethylated and 2,300 were hypomethylated (Cheng et al., 2018). In contrast, a global increase in DNA methylation was found during orange fruit ripening, which led to the repression or activation of hundreds of genes. The application of DNA-methylation inhibitor interfered with orange fruit ripening, indicating that DNA hypermethylation is essential for proper ripening (Huang et al., 2019). Increased methylation activity during fruit ripening has also been found in apple (El-Sharkawy et al., 2015).

The activator PpERF61 promotes volatile linalool synthesis in peach fruit by directly binding to DRE/CRT elements on the promoters of *PpTPS1/3*. Ripening-induced expression of *PpERF61* was associated with DNA demethylation of its promoter (Wei et al., 2022). AP2/ERFs are sensitive to methylation at their binding sites (O’Malley et al., 2016) but overall, the role of DNA methylation in AP2/ERF regulation remains poorly understood. Considering the prevalence of changes in DNA methylation during fruit ripening and the extensive regulation by AP2/ERF of ripening genes, it is reasonable to assume that DNA methylation plays a vital role in the AP2/ERF regulation mechanism, but further study is required.

Post-translational modifications of histones, including methylation, acetylation, phosphorylation and ubiquitination, regulate gene expression by affecting chromatin conformation. Increasing evidence suggests that histone modifications play an important role in fruit-ripening regulation (Li et al., 2022). Studies of several fruit crops have found that histone modifiers are preferentially or specifically expressed in fruit and perform phasic differences, suggesting that they are involved in the regulation of fruit development (Aiese Cigliano et al., 2013; Xu et al., 2015; Gallusci et al., 2016). The acetylation status of histones has been best studied. This is a reversible mark regulated by histone acetyltransferases and histone deacetylases, usually related to genes’ transcriptional activity (Shen et al., 2015).

In tomato fruit, inhibition of *SlHDA3* or *SlHDA1* expression causes significant upregulation of several ethylene-biosynthesis genes and cell wall-modification genes, resulting in accelerated ripening and reduced storability (Guo et al., 2017b,^2018^). However, inhibition of *SlHDT3*, which belongs to the HD2 family of histone deacetylases, has the opposite effect (Guo et al., 2017a). Recent studies have found that histone modifiers, such as histone deacetylases, are recruited by AP2/ERFs in fruit-ripening regulation. In apple, MdHDA19 is recruited to the MdERF4–MdTPL4 complex and inhibits fruit ripening by reducing the expression of *MdACSa* (Hu et al., 2022a). In tomato, the SlERF.F12–TPL2–HDA1/HDA3 protein complex represses the expression of ripening-related genes, including *ACS2*, *ACS4*, *PG2a*, and *PL*. These examples of transcriptional repression rely on reduced levels of histone acetylation marks H3K9Ac and H3K27Ac in the promoter regions of the target genes (Deng et al., 2022). In banana, MaERF11 recruits MaHDA1 to repress the expression of ethylene-biosynthesis and cell wall-degradation genes (Han et al., 2016). As already noted, histone deacetylases play an essential role in the EAR motif-mediated mechanism of transcriptional repression.

MicroRNAs (miRNAs) are an important class of endogenous small RNAs in plants; they target mRNA through complementary base pairing and induce gene silencing by inhibiting translation or initiating mRNA degradation. The miRNAs are essential regulators, at the post-transcriptional level, of various biological processes in plants, such as development and stress responses (He et al., 2022). Mature miRNAs are usually 20–24 nucleotides in length, and differences in length lead to distinct functions (Manavella et al., 2012). MiR172–AP2 is a conserved miRNA-target module in plants. A previous study in *Arabidopsis* found that miR172 specifically targets the mRNA of AP2 (Chen, 2004). There is increasing evidence that this module plays a crucial role in fruit ripening. In tomato, miR172 specifically targets *SlAP2a*. Overexpression of *miR172* represses *SlAP2a* expression, resulting in enhanced ethylene biosynthesis and coloration (Chung et al., 2020). In apple, MdAP2_1a, targeted by miR172, transactivates *MdMYB10* and positively regulates anthocyanin biosynthesis. Overexpression of *miR172* represses the expression of *MdAP2_1a*, thereby inhibiting anthocyanin accumulation (Ding et al., 2022). A study on an apple breeding population revealed a transposon insertional allele of miR172 with reduced *miR172* expression which was associated with large fruit. Overexpression of *miR172* negatively affected fruit development and fruit size (Yao et al., 2015). Moreover, in *Arabidopsis*, AP2 positively regulated *miR156* but negatively regulated *miR172*, and both miRNAs influenced *AP2* expression, indicating that AP2 and miRNAs have complex direct feedback loops in plants (Yant et al., 2010).

Plant genomes encode a considerable number of long non-coding RNAs (lncRNAs)—usually over 200 nucleotides in length with no discernable coding potential—which play an important role in essential biological processes (Chekanova, 2015). The lncRNAs affect all elements of genes, such as promoters, untranslated regions, exons, introns, and terminators, and control gene expression at the levels of chromatin accessibility, transcription, splicing, and translation (Wierzbicki et al., 2021). In apple, the lncRNA MdLNC499, located near *MdERF109*, was identified as a *cis*-regulator of *MdERF109* expression by Promoter:β-*glucuronidase* reporter analysis and Hi-C sequencing. Promoter *cis*-element analysis found the presence of a W-box element in *MdLNC499* promoter, which is regulated by MdWRKY1. The MdWRKY1–MdLNC499–MdERF109 transcriptional cascade was reconstructed in apple fruit and callus by transient expression and stable transformation. MdERF109 promotes coloration by directly binding to anthocyanin-related gene promoters, including *MdCHS*, *MdUFGT*, and *BASIC HELIX–LOOP–HELIX 3* (*MdbHLH3*) (Ma et al., 2021).

## Conclusion and perspective

**FIGURE 2:**
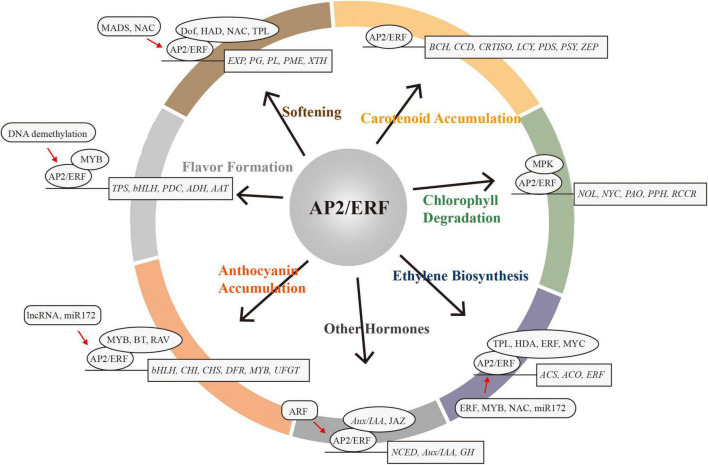
The AP2/ERF superfamily acts as a critical regulator in fruit ripening. Target genes are in rectangles. Protein–protein interactions or co-regulators are in ovals. Red arrows represent expression regulation by AP2/ERF.

The AP2/ERF superfamily is a large class of transcription factors in plants that exhibit coordinated changes in expression during fruit ripening. AP2/ERFs play critical regulatory roles in intrinsic and extrinsic quality development during fruit ripening (Figure 2). However, our understanding of the molecular mechanisms of AP2/ERF is still limited, especially with respect to the identification of co-regulators, and the influence of post-translational and epigenetic modifications. With the application of modern molecular biology and high-throughput sequencing technology, such as chromatin immunoprecipitation, DNA affinity purification sequencing, immunoprecipitation coupled with mass spectrometry analysis, next-generation sequencing, genome-wide association studies, the new gene-editing system CRISPR/Cas9, and so on, future dissection and exploration of the AP2/ERF regulatory network will broaden our understanding of quality formation during fruit ripening. In the 1990s, great effort was made to improve tomato storability by deciphering key genes in ethylene synthesis. Today, functional analyses of many ripening-related AP2/ERFs has allowed us to regulate the formation and maintenance of specific quality traits more precisely, while avoiding the interference of other, unexpected quality traits. In the future, altering the function and expression of specific AP2/ERF family members through synthetic biology techniques will provide new approaches to improving internal and external fruit quality and storability.

## Author contributions

HM planned the review manuscript. YZ, ZF, YC, XG, SC, and HM prepared the manuscript. All authors have read and approved the manuscript for publication.
